# The Boron Advantage: The Evolution and Diversification of Boron’s Applications in Medicinal Chemistry

**DOI:** 10.3390/ph15030264

**Published:** 2022-02-22

**Authors:** Katia Messner, Billy Vuong, Geoffrey K. Tranmer

**Affiliations:** 1 Rady Faculty of Health Science, College of Pharmacy, University of Manitoba, Winnipeg, MB R3E 0T5, Canada; messner1@myumanitoba.ca (K.M.); vuongb@myumanitoba.ca (B.V.); 2 Department of Chemistry, Faculty of Science, University of Manitoba, Winnipeg, MB R3E 0T5, Canada

**Keywords:** boron, medicinal chemistry, diazaborines, boronic acids, boron clusters, carboranes, benzoxaboroles, bortezomib

## Abstract

In this review, the history of boron’s early use in drugs, and the history of the use of boron functional groups in medicinal chemistry applications are discussed. This includes diazaborines, boronic acids, benzoxaboroles, boron clusters, and carboranes. Furthermore, critical developments from these functional groups are highlighted along with recent developments, which exemplify potential prospects. Lastly, the application of boron in the form of a prodrug, softdrug, and as a nanocarrier are discussed to showcase boron’s emergence into new and exciting fields. Overall, we emphasize the evolution of organoboron therapeutic agents as privileged structures in medicinal chemistry and outline the impact that boron has had on drug discovery and development.

## 1. Introduction

In recent years, boron-containing compounds have emerged as a new and exciting field of research in medicinal chemistry [1]. Boron is a metalloid element with unique characteristics that make it an appealing candidate in medicinal chemistry [2]. At physiological pH, boron has a vacant p orbital in its stable, neutral sp^2^ hybridized form that can readily accept an electron pair. This makes boron very electrophilic, allowing it to act as a Lewis acid and convert to an anionic sp^3^ hybridized form upon accepting an electron pair [3]. This quality makes boron attractive to use in drug candidates as it can form coordinated covalent bonds through nucleophilic occupation of its empty p orbital [4]. Through the modification of the molecule, this quality can be adjusted to fit the needs of a drug [5].

There are very few compounds in nature that have boron incorporated into the structure. These include peptidic antibiotics, borophycin, aplasmomycin, tartolon B, and boromycin [6]. As a result of this, medicinal chemists do not have many natural models to act as a template for drug design. As for whether boron is toxic, studies that were conducted in the early 20th century have reported that doses of 77–699 mg/day of boric acid could potentially be harmful to humans [7]. More recently however, boron has been found to be a known essential nutrient in plants and animals/humans, although its physiological functions are poorly understood [8]. In fact, the World Health Organization (WHO) suggests an intake of 0.4 mg B/kg body weight/day for humans [8]. In cases of acute ingestions of boric acid (10 mg to 88.8 g) in 784 patients, the majority (88.7%) were asymptomatic, with symptomatic patients experiencing vomiting, abdominal pain, and diarrhea as the most common adverse effects [9].

A critical development that lead to the growth of boron medicinal chemistry [10] is the Suzuki–Miyamura coupling reaction [11,12]. This reaction was a critical discovery for boronic acid chemistry as it increased research focus on boronic acids. First reported in 1979, the Suzuki–Miyamura coupling reaction is a carbon-carbon bond forming reaction between an alkenyl borane or catecholate and an aryl halide in the presence of a base, and catalyzed with palladium [11]. The reaction has been improved since then, with conditions that have been optimized for different catalysts and ligands, bases, solvents, and additives [11,12]. The greater interest in boron as a reagent for this reaction has led to more discoveries and interest in boron medicinal chemistry over the years [4].

**Figure 1:**
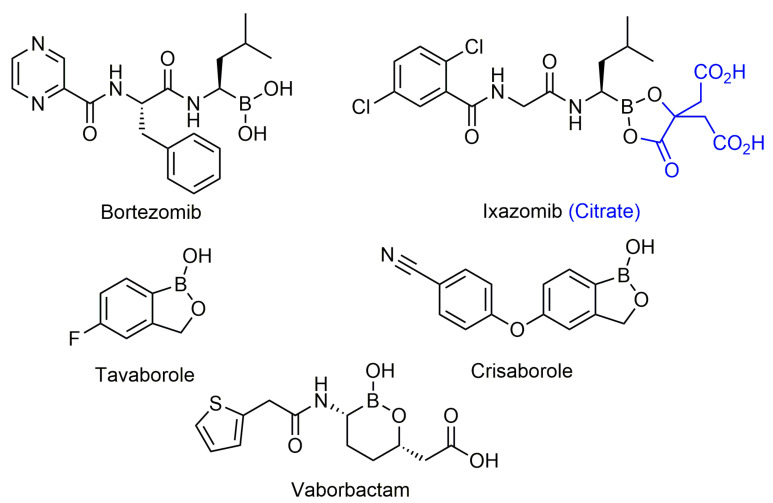
FDA-approved boron-containing drugs.

Within the last two decades, there have been major advances in boron organic chemistry, with new methods and new catalysts discovered. This has made the incorporation of boron functional groups into drugs more readily accessible and practicable [13]. The increasing consideration of boron in drug design has led to successful expansion of boron-containing compounds in recent years. Notably, five FDA-approved boron-containing compounds have been developed: bortezomib (Velcade), tavaborole (Kerydin), ixazomib (Ninlaro), crisaborole (Eucrisa), and vaborbactam (in combination with meropenem in Vabomere), (Figure 1) which have drawn considerable attention to the use of boron as a viable candidate for further drug development.

In this article, we will discuss applications of boron-containing compounds from past to present, focusing on critical developments including the development of core boron groups that are used in drug design today, along with recent developments and discoveries in the field. The boron groups that are discussed will be of the two branches of boron medicinal chemistry: (1) single boron compounds, and (2) boron clusters [6]. We will also discuss the recent developments of utilizing boron functional groups as prodrugs, softdrugs, or as various carrier groups (liposomes, vesicles, nanoparticles, etc.), to increase drug bioavailability. Boron has a plethora of potential therapeutic applications and will continue to develop into a very important field in medicinal chemistry and as a critical tool in developing new approaches to act on novel targets for treating diseases.

## 2. Boron Medicinal Chemistry

### 2.1. The Early History of Boron Medicinal Agents

**Figure 2:**
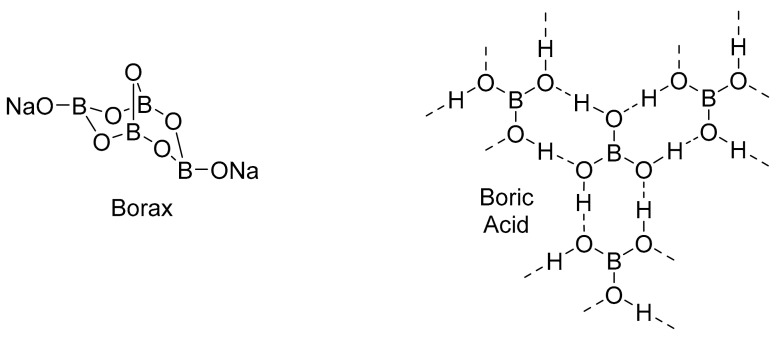
The earliest boron medicinal agents, borax, and boric acid.

Boron has a long history of use within human society. Boron may have first been used thousands of years ago in ancient Babylonian and Egyptian societies in the form of borax [14]. However, the first definitive evidence of boron being used was in the form of mineral borax (Figure 2), tincal that was imported to Mecca, Medina, and China by Arab transporters [14]. In the 18th century, boric acid (Figure 2) was made and used as a mild antiseptic and eye wash [15]. In 1857, boron was discovered in plants, and shortly after in the 1870′s, sodium borate and boric acid were discovered to act as preservatives [7]. Boron as a preservative was very successful for the next 50 years, as it was used for meat and dairy preservation during the first and second world wars. However, shortly after these wars there was a developing belief that boric acid was harmful and was no longer used as a preservative [7]. During this time, a critical discovery led to the resurface of boron in science: boron neutron capture therapy (BNCT).

### 2.2. Boron Neutron Capture Therapy

**Figure 3:**
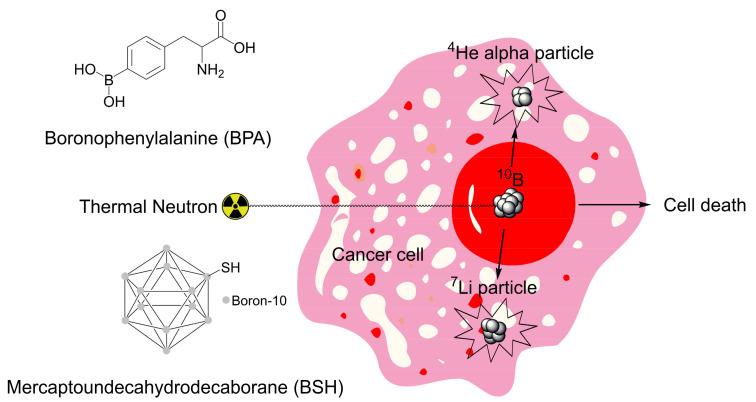
Boron neutron capture therapy (BNCT) agents boronophenylalanine (BPA) and mercaptoundecahydrodecaborane (BSH) with ^10^B isotope. ^10^B captures low energy thermal neutrons from radiation therapy and splits into a recoiled ^7^Li particle and a ^4^He α-particle, damaging the cancer cell resulting in cell death.

The utilization of boron in radiation therapy for cancer has been proposed as early as the mid-20th century, where a stable, non-radioactive isotope of boron (^10^B) was used. Boron neutron capture therapy (BNCT) is a technique that is used to treat cancer that involves radiation therapy with low energy neutrons (thermal neutrons) that become captured by ^10^B nuclei, splitting it into an alpha particle (^4^He) and a recoiled ^7^Li nuclei, both of which are high energy [16]. Both resultant particles are unable to travel far in tissue and undergo short, linear trajectories within the confines of a cell [17,18]. For BNCT to be an effective cancer treatment, ^10^B-containing molecules must concentrate within cancerous cells. Currently, boronophenylalanine (BPA, Figure 3) and mercaptoundecahydrodecaborane (BSH, Figure 3) are two ^10^B-containing molecules that have undergone clinical trials with BNCT for high grade gliomas, melanoma, hepatic metastasis, and recurrent head and neck tumors [17]. Initially, the single boron-containing BPA was used for BNCT, however, boron and carborane polyhedral cages were synthesized in the 1960s [19] that allowed for multiple boron atoms to be transported into the cell. This led to an increased cellular uptake of boron and thus mercaptoundecahydrodecaborane (BSH) was synthesized as a multiple boron-containing drug for BNCT [16]. BPA is actively taken up by cancer cells through L-type amino acid transporter 1, which is highly upregulated in several cancers [20], whereas BSH is typically used for malignant gliomas and accumulates in areas where the blood brain barrier is compromised by the cancer [21].

### 2.3. Diazaborines

Diazaborines are a six-membered heterocycle ring containing a boron, two nitrogen, and three carbon atoms that were first described for their antimicrobial properties in the 60s [22]. In 1980, diazaborines were first described to induce morphological changes to bacteria resulting in abnormal growth [23]. Further studies with 1,2-dihydro-1-hydroxy-6-methyl-2-(propanesulphonyl)-thieno (3,2-D) (1,2,3)-diazaborine (Table 1) believed that it functioned to inhibit lipopolysaccharide (LPS) synthesis, a key component of the Gram-negative bacterial cell wall [24,25]. Additionally, it was found that diazaborines had activity against *Enterobacter*, *Neisseria Gonorrhoeae, Proteus,* and *Salmonella*, suggesting that diazaborines may have potential as a broad-spectrum antibiotic [22]. It was not until years later that the mechanism in which diazaborines inhibit bacterial growth was found to be through the inhibition of NAD(P)H-dependent enoyl acyl carrier protein reductase (ENR), which catalyzes the last step of fatty acid synthase [22]. Specifically, the boron atom in the diazaborine forms a covalent adduct with the 2′-hydroxyl of the nicotinamide ribose moiety [26].

Recently, the application of diazaborines has been expanded to other targets such as *Mycobacterium tuberculosis*, which has a similar enzyme (InhA) to ENR. It was anticipated that diazaborines would inhibit InhA, however it was not as effective as the antitubercular prodrug isoniazid (INH) but had greater activity than pyrazinamide, another antitubercular [27]. AN12855 (Table 1), a recently developed diazaborine InhA inhibitor that binds to both the cofactor and substrate-binding unit of InhA, was effective in acute and chronic models of tuberculosis and was able to act upon INH-resistant *M. tuberculosis* strains [28]. In vivo studies of AN12855 found that it was more effective than INH, making it a viable candidate for the treatment of tuberculosis [29]. Diazaborines have also been found to inhibit the growth of yeast cells [30,31]. There have also been studies of diazaborines acting as inhibitors of ribosome biogenesis through binding of AAA-ATPase Drg1, preventing ATP hydrolysis, and blocking release of Rip24 from pre-60s particles and inhibiting eukaryotic ribosome biogenesis [32]. With the recent emergence of novel diazaborines that act on a large variety of targets, there is a lot of potential for future drug development.

### 2.4. Boronic Acids

#### 2.4.1. Peptidic Boronic Acids

##### General Information and History

Peptidic boronic acids were one of the first classes of boronic acids that were described for their therapeutic properties [33,34]. Initially, boronic acids were found to inhibit several serine proteases such as chymase [34], thrombin [35], and chymotrypsin [36]. It was not until 1997 that bortezomib (Velcade) was discovered to have inhibitory effects on the 26S proteasome, a critical protein that is involved in maintaining cell homeostasis by degrading damaged or misfolded proteins. Bortezomib became the first boronic acid-containing drug to enter human trials for advanced cancer in 1998 [37], and gain FDA approval in 2003 for multiple myeloma (MM) and in 2004 for mantle cell lymphoma [38,39]. Bortezomib continues to be used as a first line therapy for MM [40] and was influential in promoting interest in boron drug discovery, in addition to becoming a template for designing compounds that inhibit the proteasome. With the success of bortezomib, boron-containing drugs became a popular target for drug development, leading to ixazomib also becoming FDA approved for multiple myeloma.

##### Bortezomib and Ixazomib

Bortezomib is an intravenously-administered drug and its mechanism of action targets the chymotrypsin-like β 5 subunit in the core 20S proteasome [38]. It binds to the nucleophilic threonine residues in the catalytic site forming a reversible covalent borate complex which inhibits proteasomal activity, resulting in cell death [39,41]. Although bortezomib has been successful in treating MM, there are several adverse effects that are associated that include drug resistance, gastrointestinal issues, and peripheral neuropathy [42]. These potential side effects have led to the development of a second generation of proteasome inhibitors that have a higher specificity for proteasomal subunit catalytic sites and less potential adverse effects. In 2015, ixazomib (Ninlaro), a second-generation boron-containing proteasome inhibitor, was FDA-approved for the treatment of MM in patients who had received at least one prior therapy. Ixazomib differs from bortezomib in that it is taken orally in the prodrug form of ixazomib citrate, undergoing hydrolysis to its activated form. Additionally, ixazomib is rapidly absorbed and showed improved pharmacokinetics and antitumor activity in preclinical studies [43]. In clinical studies, its toxicity profile showed improvement over bortezomib with lower incidences of major adverse effects such as drug resistance and peripheral neuropathy [43]. With the success of these two drugs in treatment of MM, peptidic boronic acids have become a prime candidate for drug development as proteasome inhibitors due to the covalent boron interaction with nucleophilic residues.

##### Recent Studies on Proteasome Inhibitors

The exploration of novel boronic acid proteasome inhibitors as viable cancer therapeutics is still ongoing. In 2009, tripeptide derivatives of bortezomib were investigated as a potential new class of proteasome inhibitors [44]. That same year, boronic acid derivatives of tyropeptin were synthesized, with one potential drug candidate showing a nine-fold increase in the inhibition of proteasomal activity compared to bortezomib [45]. Further studies on boronic acid tyropeptin derivatives (AS-06, AS-29, Table 2) by the same group determined that their drug candidates induced apoptosis through the caspase 8 and 9 cascades, and showed promising in vivo results in supressing tumor growth in xenograft mice with MM [46]. Since then, several novel boronic acid protease inhibitors have been developed with comparable in vitro and in vivo results to bortezomib [47,48]. In addition to cancer, boronic acid proteasome inhibitors have also been studied for other therapeutic applications as well, such as treatment for malaria [49]. Through the screening of a library of human boronic acid proteasome inhibitors, one study identified several potential candidates that showed preferential selectivity for the *Plasmodium falciparum* proteasome with weaker, reversible activity against human cancer cell lines [50]. These drug candidates can serve as a template for future development of antiplasmodial drugs that can provide maximal efficacy against target parasites while having minimal effects in humans.

##### Other Applications of Peptidic Boronic Acids

In 2013, Branched peptidic boronic acids were developed and found to bind to HIV-1 RNA, with the boronic acid moiety enhancing the compound RNA binding ability [51]. Later studies found that the branched peptide inhibited HIV-1 p24 production and HIV replication in vitro [52]. These branched, peptidic boronic acids were also investigated for potential antimicrobial activity. They were able to potently inhibit the growth of *C. albicans*, *S. aureus*, and *E. coli* at minimum inhibitory concentration (MIC) of 1 µg/mL, and did not affect the viability of U87 CXCR4 cell line at 10-fold MIC [53]. Peptidic boronic acids were also investigated as potential hypoxia inducible factor 1α (HIF-1α) inhibitors. One compound, GN26361 (Table 2), potently inhibited the accumulation of HIF-1α under hypoxic conditions via the inhibition of hypoxia-induced HIF-1α transcriptional activity in HeLa cells (IC50 = 0.74 μM) [54]. Peptidic boronic acids have also been studied for other microbial targets including as a hepatitis C virus (HCV) NS3/4A protease inhibitor [55], an antitubercular drug [56], penicillin-binding proteins [57], histone deacetylase (HDAC) inhibitors [58], and autotaxin (ATX) inhibitors [59]. The variety of potential targets for peptidic boronic acids makes them a very attractive candidate for future drug development.

##### Peptidic Boronic Acids in Clinical Trials

Within the last decade, several peptidic boronic acids have been entered into clinical trials [13,39]. However, many promising compounds have fallen short of completing their clinical trials. PHX1149 (dutogliptin, Table 2), a dipeptidyl peptidase (DPP4) inhibitor, was reported to be in phase III clinical trials [4] and was being studied for the treatment of type 2 diabetes [60]. In 2010, all of its phase III trials were terminated after Phenomix, the biopharmaceutical company conducting the trials went out of business [61]. Recently, PHX1149 has been picked up by Recardio and re-entered clinical trials for early recovery of myocardial infarction, and is currently enrolling participants for its phase II study [62]. Another candidate PT100 (Talabostat/Val-boroPro), was in phase III clinical trials for several cancers as an inhibitor of DPP4 and fibroblast activation protein (FAP) [4]. Phase III trials were ended due to Val-boroPro’s lack of inhibition of endopeptidase activity of FAP [39]. Recently in 2020, phase II trials on talabostat and pembrolizumab for the treatment of advanced solid cancers began [63]. TRI50c (Table 2) was another candidate that was last reported to be in phase III trials as an anti-coagulant in 2009 in both oral form (TGN167) and intravenous form (TGN255) [4]. CEP-18770 (delanzomib), was also in clinical trials as a proteasome inhibitor, however its trails were terminated due to its toxicity [64]. No further updates have been reported on these drugs other than the recent reintroduction of talabostat and dutogliptin in clinical trials.

#### 2.4.2. Benzoxaboroles

##### General Information and History

Benzoxaboroles are cyclic boronic acid hemiesters called oxaboroles that are directly connected to a benzene ring. They were first synthesized by Torsell in 1957 [41,65]. These compounds are distinct as the boronic functional group is fused into a heteroaromatic ring [13], making it less susceptible to the hydrolysis of the boron-carbon bond, an issue that is commonly found in typical boronic acids [65]. In addition, they are more soluble in water than other boronic acids [65] and have been repeatedly shown to have a low intrinsic toxicity [66]. While they have many beneficial properties that make them an excellent group to incorporate into drug design, they had only been recently studied for these purposes. For 50 years since their initial discovery, benzoxaboroles had been mainly applied in organic synthesis, glycopeptide recognition, and supramolecular chemistry [67]. Benzoxaboroles in drug design gained traction in 2006, when tavaborole was found to possess potent antifungal activity [65]. This discovery led to greater study of this boron group, and it has been found to possess antimicrobial, antiviral, anticancer properties, among several others [67].

##### Tavaborole and Crisaborole

Tavaborole (Kerydin) was the first benzoxaborole to gain FDA approval in 2014 for the treatment of onychomycosis, a fungal infection [68]. Tavaborole inhibits yeast cytoplasmic leucyl tRNA synthetase (LeuRS) by forming a stable adduct with the enzyme, specifically between the boron in tavaborole and oxygen atoms in the LeuRS-editing site to prevent catalytic turnover and subsequent protein synthesis [69]. This molecular interaction in particular has become a target of interest in designing benzoxaboroles for drug activity, with many benzoxaboroles being found/designed to inhibit aminoacyl-tRNA synthetases of other organisms [70,71]. Crisaborole (Eucrisa/AN2728) was the second benzoxaborole to gain FDA approval in 2016 to treat mild to moderate eczema (atopic dermatitis) in patients two years of age and older [72]. Crisaborole functions as a phosphodiesterase-4 (PDE4) inhibitor and displays inhibitory activity against the release of inflammatory cytokines [73]. Although the exact mechanism of which crisaborole inhibits PDE4 is poorly understood, its success is an excellent example of the therapeutic potential of benzoxaboroles.

##### Benzoxaboroles as Antitubercular, Antitrypanosomal, and Antimalarial Agents

Along with the two FDA-approved benzoxaboroles, there are many other promising candidates in clinical and preclinical trials. GSK3036656 (Table 3) is an *M. tuberculosis* LeuRS inhibitor that will soon be entering phase II trials [74]. Its mechanism is similar to tavaborole, with the boron forming an adduct with the terminal nucleotide of tRNA, leading to the formation of a complex that eventually inhibits protein synthesis [65]. This drug, in particular, is very promising as it shows potent inhibition in the mid nanomolar range [67] and is highly selective against human mitochondrial and cytoplasmic LeuRS [71]. Phase I trials of the drug further proved its potential as it was safe and generally well tolerated after single and multiple doses and has a short duration of treatment. [75] What is of interest to note, is that dimeric benzoxaboroles showed increased inhibitory activity compared to monomeric benzoxaboroles through a non-LeuRS interaction, which could potentially overcome bacterial resistance that is associated with monomeric benzoxaboroles [76].

Benzoxaboroles have been proposed as potential anti-trypanosome candidates, with acoziborole (AN5568/SCYX-7158, Table 3) currently in clinical trials for the treatment of Human African Trypanosomiasis, (sleeping sickness). Acoziborole kills trypanosomes through the inhibition of trypanosomal mRNA-processing endonuclease cleavage and polyadenylation specificity factor subunit 3 (CPSF3), which is involved in processing parasitic mRNA [77]. Acoziborole is taken orally, has high bioavailability across species, and is able to cross the blood brain barrier and reach therapeutic concentrations in in vivo rodent studies [78]. This makes acoziborole of particular significance over current treatments that have issues with low efficacy in late stage treatment, high toxicity, and resistance potential, which resulted in the need of new drug candidates for treating this neglected disease [78,79]. Currently, acoziborole is in phase II/III trials, and scheduled to be completed at the end of 2020 [80]. In addition to acoziborole, several other benzoxaboroles have been explored as potential antitrypanosomals [81,82]. These benzoxaborole-derived drugs have also shown promise in treating livestock, in which trypanosomiasis can be particularly devastating [83].

Benzoxaboroles have also shown promise as antimalarial drugs. A potent antimalarial, AN3661 has shown potent activity (IC_50_ 32-64 nM) against *P. falciparum* strains in vitro and ex vivo, with CPSF3 identified as its target receptor [84]. AN3661 (Table 3) was also found to be effective against multi-drug resistant laboratory strains of *P. falciparum* [84] and is now a preclinical candidate [67]. Several [85,86] other antimalarial benzoxaboroles have been developed and display excellent in vitro potency and selectivity, pharmacokinetic properties, and in vivo efficacy, with both LeuRS and CPSF3 as potential targets [87]. The results of these studies have shown that benzoxaboroles as antibacterial, antiparasitic, and antimalarial prophylactics have the potential for further drug development.

##### Miscellaneous Applications of Benzoxaboroles

Benzoxaboroles have also been designed to act on a variety of different targets for various physiological conditions. Benzoxaboroles have recently been investigated as anticancer agents, with some candidates showing potent activity against ovarian cancer cells in the nanomolar range in vitro, and adequate efficacy in vivo in SKOV3 mouse xenograft models [88,89]. Although the molecular target of these anticancer benzoxaboroles is not defined, the authors determined that LeuRS is not the molecular target but potentially carbonic anhydrase (CA) [89]. In recent years, benzoxaboroles have been further investigated as CA inhibitors [90,91]. In 2019, a dimeric benzoxaborole with a glutamic acid central core was found to be a potent inhibitor of CA IX, a tumor-associated isoform of CA [92]. In 2018, a group of benzoxaboroles were investigated for their ability to treat cutaneous leishmaniasis with two effective compounds, one that was administered through topical application and the other through oral administration [93]. Benzoxaboroles also have potential to be effective antibiotics, with one study examining their activity against *S. pneumoniae* relative to clarithromycin, an antibiotic that is active against Gram-positive strains [94]. Benzoxaboroles have also been explored for anti-inflammatory activity, with some candidates inhibiting the release of proinflammatory cytokines [95,96,97] and others acting through the inhibition of PDE4 [98], similar to crisaborole. Benzoxaboroles have also been studied as HCV NS3 protease inhibitors [99], β-lactamase inhibitors [100], and as LeuRS inhibitors [101]. Benzoxaborole AN7973 has been investigated as a treatment for cryptosporidiosis, which causes life-threatening diarrhea and whose only treatment option is ineffective in malnourished children and immunocompromised individuals [102]. AN7973 (Table 4) was initially proposed for cattle trypanosomiasis [83], but was later replaced and has been found to be effective for treating cryptosporidiosis. Although the exact mechanism is unknown, AN7973 appears to be parasiticidal by inhibiting intracellular parasite development, and was effective against the two cryptosporidium species that were most relevant to humans in vivo [102]. It was able to reduce the severity of a major symptom (dehydration), and was efficacious in immunocompromised mice, making AN7973 a promising alternative treatment for cryptosporidiosis [102]. The variety of applications of benzoxaboroles that are provided show that they are worth further investigation as novel therapeutics.

##### Benzoxaboroles in Clinical Trials

Although two of the five FDA-approved boron-containing drugs are benzoxaboroles, there have been several compounds that made it to clinical trials. AN2898 (Table 4), a PDE4 inhibitor, was in phase II clinical trials for atopic dermatitis and psoriasis alongside crisaborole in 2011 [103]. Since then, there have not been any further updates. AN2718, a treatment for onychomycosis that is similar to tavaborole, was last reported to be in phase I trials over 10 years ago with no further updates [104]. AN3365 (GSK2551052/epetraborole, Table 4), a LeuRS inhibitor, was in clinical trials for the treatment of urinary tract infections [6] and made it to phase II trials before drug resistance was observed and it was discontinued [66,105]. Although the development of benzoxaboroles as therapeutics is relatively new, their flexibility to act on a variety of different targets such as LeuRS, PDE4, CPSF3, and their anti-inflammatory activity shows their potential as excellent pharmacophores that warrants further consideration.

#### 2.4.3. Other Boronic Acids

Although many of the successful boronic acids fall into the peptidic boronic acids or benzoxaboroles categories, there are other boronic acid compounds that have shown potential in recent years, such as the β-lactamase inhibitor vaborbactam. In 2017, vaborbactam, in combination with meropenem as Vabomere, was FDA-approved for the treatment of urinary tract infections [106]. This FDA approval was significant as vaborbactam was the first boronic acid β-lactamase inhibitor that was approved, and was a culmination of over 40 years of study [57,100,107]. In this section, boronic acids with novel applications and structures will be discussed.

##### Cyclic Boronic Acids as β-Lactamase Inhibitors

Cyclic boronates that function as β-lactamase inhibitors have recently gained attention after the FDA approval of vaborbactam. β-lactamases are bacterial enzymes that provide multi-drug resistances to β-lactam-based antibiotics through the hydrolysis of the β-lactam ring. Boronic acid–based β-lactamase inhibitors are not based on the structure of the substrate β-lactam, but rather are transition state analogs, making them less sensitive to resistance mechanisms or mutations such as enzyme upregulation and porin channel deficiencies [107,108]. Boronic acids inhibit β-lactamases using boron’s empty p orbital to form a covalent bond with the catalytic serine residue of the β-lactamase, mimicking the transition state, and forming a serine trap [100]. This trap is stable long-term relative to other β-lactam-based inhibitors, which are typically hydrolyzed and diffuse away [109]. The first study that determined boronic acids as β-lactamase inhibitors was conducted by Keener and Waley, who discovered that boric acid, phenylboronic acid, and *m*-aminophenylboronic acid weakly inhibit class A β-lactamase from *Bacillus cereus* [110]. From this discovery, boronic acids were proposed and further studied as potential treatments to combat β-lactam resistant infections [110]. Boronic acids have been found to inhibit (1) Ambler [111] class 1 serine hydrolases: class A [108,112], class C [112,113], class D β-lactamases [114,115,116], and (2) metallohydrolases: class B β-lactamases [115,116], or (3) can act on multiple classes [112,114,116]. Vaborbactam, Taniborbactam, and QPX7728 are the lead compounds in this area, making them potentially useful as a combination therapy with other β-lactam antibiotics to treat antibiotic-resistant bacterial infections.

##### Vaborbactam, Taniborbactam and QPX7728

Vaborbactam (RPX7009) is the first and only FDA-approved cyclic boronic acid β-lactamase inhibitor. It is used in combination with the antibiotic meropenem as Vabomere and is used to treat urinary tract infections. Vaborbactam is highly active in class A and C β-lactamases including the *Klebsiella pnuemoniae* carbapenemase (KPC) β-lactamase [117,118]. Due to vaborbactam having a weak effect against class B or D β-lactamases, it is ineffective in restoring antibiotic activity against multidrug-resistant nonfermenting Gram-negative bacilli [117]. In the case of Vabomere, vaborbactam is combined with meropenem so that it can restore its activity against KPC β-lactamase-producing, carbapenem-resistant, *Enterobacteriaceae* [118]. In addition, vaborbactam is not metabolized in vivo [117] and there has yet to be resistance due to mutations in β-lactamase genes reported [119].

Broad spectrum β-lactamase inhibitors that show potential are taniborbactam (VNRX-5133) and QPX7728, Table 5. Taniborbactam will be entering phase III clinical trials in combination with cefepime for the treatment of complicated urinary tract infections and hospital-acquired or ventilator-associated bacterial pneumonia [120]. It is the first β-lactamase inhibitor that has high inhibitory activity against Ambler class A, B, C, and D β-lactamases [120]. In serine hydrolases, it acts as a reversible covalent inhibitor with slow dissociation (t_1/2_ = 30–105 min) from the active site, while in metallohydrolases, it acts as a competitive inhibitor [120]. QPX7728 is another broad-spectrum inhibitor of serine and metallo β-lactamases that is able to inhibit class A, B, C, and D β-lactamases in the nanomolar range [121]. Against class A and C β-lactamases, QPX7728 has comparable IC_50_ values to FDA-approved avibactam, relebactam, and vaborbactam, while also being able to inhibit class D carbapenemases from *A. baumanii*, which taniborbactam is unable to do [121]. Due to its effectiveness against all classes of β-lactamases, taniborbactam and QPX7728 have the potential to become broad range β-lactamase inhibitors to use against bacteria that are resistant to β-lactam antibiotics.

##### Additional Therapeutic Applications of Boronic Acids

Boronic acids have also been investigated for a variety of different applications [122]. Boronic acids have been explored as potential HDAC inhibitors, but with undesirable pharmacokinetic profiles they were not further developed [39]. Additionally, α-amino cyclic boronates have been designed as inhibitors of the HCV NS3 protease, utilizing the covalent boron interaction with the catalytic serine residue with sub micromolar IC_50_ [123]. Recently, anti-inflammatory oxazaborine compounds have been proposed to target the NLRP3 inflammasome [124]. The boron-containing compounds were compared to their respective carbon analogues which were not as effective, suggesting that boron plays a critical role in the inhibition process [124]. In a different study, a series of boronic acid P2X7R inhibitors as anti-inflammatories were able to inhibit P2X7R through antagonistic mechanisms, outperforming other P2X7R inhibitors such as BBG and A740003, at reducing P2X7R activity and inflammation [125]. In another study, SX-517 (Table 5) was the first reported boronic acid chemokine antagonist for CXCR 1 and 2 and was able to significantly inhibit inflammation in vivo [126]. SX-517 was eventually replaced with a new lead compound SX-576 (Table 5), which had greater systemic exposure, greater inhibition in vitro, increased metabolic stability, and improved pharmacokinetic properties in vivo [127]. Lastly, a structure-activity relationship (SAR) study was conducted to create an orally available compound with improved aqueous solubility with the same activity level as SX-576 [128]. The third-generation compound that was synthesized not only met the desired results, it was also able to significantly reduce the influx of neutrophils [128]. Boronic acid-substituted stilbenes have been investigated as ligands for transtherytin (TTR) to stabilize the homotetramer, preventing fibril formation that leads to amyloidosis [129]. The boronic acid moiety is key to forming a reversible covalent interaction with a serine residue in combination with acting as a TTR ligand, enhancing stabilization of the homotetramer, and preventing aggregation of TTR monomers that would lead to amyloidosis [129]. In addition to bortezomib and ixazomib, non-peptidic boronic acids as proteasome inhibitors that have better chemical stability, immunogenicity, and improved membrane permeability were proposed, with five compound’s inhibition of proteasome activity residing in the nanomolar range that was comparable to that of bortezomib [130]. Other boronic acids have been proposed as: anti-MRSA (methicillin-resistant *Staphylococcus aureus*) antibiotics [131], ATX inhibitors [132], NorA efflux pump inhibitors [133], and HIV-1 integrase inhibitors [134].

### 2.5. Boron Clusters

#### 2.5.1. General Information and Chemistry of Boron Clusters

Boron clusters are polyhedral boranes, consisting of boron and hydrogen that form a full cage (*closo*) or a nest (*nido*) structure. There are also derivatives of these clusters which include carboranes [135], which have one or more carbon-hydrogen units; heteroboranes, which are carboranes coordinated with a nonmetal; and metallocarboranes, which are carboranes that are coordinated with a metal [3]. In boron clusters, the hydrogen atoms on the boron atom are negatively charged, therefore. they are able to form dihydrogen bonds with protic hydrogen atoms [136]. The hydride character of the hydrogen atoms also prevents the formation of conventional hydrogen bonds, resulting in these 3D compounds having various degrees of hydrophobicity [137]. Boron clusters are also unique in that they are abiotic and unfamiliar to most living organisms, potentially possessing greater stability and pharmacokinetic properties than organic molecules [138]. The most studied group of boron clusters is the carboranes, specifically those with two BH units that are replaced by two CH units. These carboranes are also known as dicarba-closo-dodecaboranes and have three isomers: ortho, para, and meta [139]. Unlike the carboranes with only one CH unit, these carboranes can be used and have been used as hydrophobic pharmacophores [18]. In this section, carboranes will refer to the carboranes with two CH units. One of the most important features of carboranes is their ability to enter substitution reactions without degradation of the cage [140]. Additionally, carboranes are very chemically and thermally stable [141] and have well established chemistry that is critical to allowing exploration as a novel hydrophobic pharmacophore.

#### 2.5.2. The History of Boron Clusters

The concept of boron clusters began in 1954 when Lipscomb and colleagues described the theoretical prediction of an icosahedral borane B_12_H_12_ [142]. This theory was brought to fruition with the synthesis and isolation of the icosahedron B_12_H_12_^2−^ [143]. The first in vivo study was conducted in 1961 with Na_2_B_10_H_10_ as a boron source for BNCT [15]. This led to further applications of boron clusters in BNCT and made BNCT a more viable therapeutic option due to more ^10^B atoms being introduced into tumor cells at a given time. This was followed by the first synthesis of carboranes in 1963 [144]. By 1977, the first use of a closo-carborane group for ligand-receptor interactions was conducted [15]. The next critical advancement of boron clusters were studies describing a structure-based design of closo-carborane derivatives of estrogen [145] and retinoic acid [146]. These progressions led to what can be described as a second type of boron cluster chemistry that involves polyhedral boranes and its derivatives being used for drug delivery and design and as enriched boron compounds for BNCT [147], both which are possibilities that have only recently been explored in medicinal chemistry. In this section, boron clusters of the second type will be discussed specifically in their BNCT applications and their use as a hydrophobic pharmacophore.

#### 2.5.3. Boron Clusters in Novel BNCT Delivery Applications

Although it has been over 80 years since BNCT was first theorized [16], it is still not available today for cancer treatment. This is mainly due to the difficulty in synthesizing a BNCT agent that is both efficacious and able to achieve sufficient cellular concentrations in tumour cells that are required to treat cancer effectively [147]. In recent years, several novel boron clusters were synthesized and the delivery systems were explored to combat these issues. One approach involves using a liposome delivery mechanism to encapsulate closo-dodecaborate [148]. Interestingly, it was found that the use of spermidinum (spd) as a counter ion was essential to create high boron-containing liposomes and to deliver them efficiently to the tumour sites [148]. Additionally, mice that were treated with spd-[10BSH]-encapsulated liposomes at a dose of 30 mg had a 100% survival rate up to 100 days after thermal neutron irradiation [148]. In 2006, a unique approach to liposome delivery included incorporating ^10^B into the liposome bilayer instead of in the inner cell, creating the potential for BNCT agents and other drugs to be encapsulated and delivered to cells [149]. Another study that was conducted created liposomes that contained carboranes within their structure but was unsuccessful due to its inability to reach critical hypoxic regions of tumours [150]. In 2012, a study on BSH-encapsulating 10% distearoyl boron lipid liposomes was conducted as a novel approach to increase the boron content that was delivered to tumour cells [151]. The in vivo studies found that mice that were dosed with 15 mg B/kg had resulted in complete reduction of the tumor three weeks after thermal neutron irradiation [151]. In 2019, Asialoglycoprotein receptor-targeted micelles containing carboranes were designed for BNCT of hepatocellular carcinoma [152]. These micelles displayed no systemic toxicity in mice, had a higher accumulation of ^10^B than BSH in tumours, and increased the apoptotic rate when irradiated by thermal neutrons when compared to the BSH group [152]].

In addition to liposomes and micelles, other delivery agents for BNCT have been explored. Recently in 2020, boron-containing vesicles that were comprised of a carborane-containing polymer that could self-assemble were synthesized for BNCT [153]. They were found to accumulate within tumors for at least 24 h, were quickly taken up by tumor cells, and were stable within the bloodstream [153]. Also in 2020, a study containing deoxygalactosyl-modified carborane groups were introduced to a gastrin-releasing peptide receptor (GRPR)-selective ligand, achieving 80 boron atoms loaded per molecule [154]. To prevent uptake into the liver cells, L-deoxygalactosyl-modified carborane building blocks were used which prevented the undesired uptake by asialoglycoprotein receptors in the liver cells compared to D-deoxygalactosyl, serving as a potential model for further developments of peptide-based boron delivery systems [154]. Although the development of a suitable BNCT agent is still in the early stages, current approaches for enhancing the uptake and concentration of boron have shown the potential for the development of a viable candidate in the future. 

#### 2.5.4. Carboranes as Steroidal Agonists, Antagonists and Modulators

Since carboranes are hydrophobic pharmacophores, many have been designed to bind to steroidal receptors such as those for estrogen, androgen, and progesterone. The most well-studied carboranes are those that act as agonists, antagonists, or modulators of estrogen. In 1999, Endo et al. designed and synthesized a carborane estrogen agonist and provided proof of concept that carboranes can be used in biologically-active molecules as hydrophobic pharmacophores [155]. The study found that one of the carboranes that was synthesized could bind the estrogen receptor (ER) with stronger interactions than the natural agonist 17 β-estradiol [155]. That same year, Endo et al. reported the design of estrogen antagonists which consist of a carborane acting as the hydrophobic component [145]. Shortly after, BE120 (Table 6) was synthesized as a carborane-containing estrogen receptor agonist that had 10 times more potent activity than 17 β-estradiol, and was able to recover uterine weight and bone loss in mice [156]. Afterwards in 2009, the next generation carborane estrogen agonist BE360 (Table 6) was synthesized as a selective estrogen receptor modulator (SERM) with several advantages over BE120, Table 6 [157]. These include restoring bone loss in vivo from estrogen deficiency in both ovariectomized and orchidectomized mice with no androgenic effect in the sex organs, and unlike BE120, BE360 prevented estrogenic activity on the uterus, reducing the risk of uterine cancer [157]. These advantages over traditional estrogen treatment make BE360 an attractive candidate for the treatment of individuals with postmenopausal osteoporosis [157]. In addition, BE360 has also been studied for its ability to treat postmenopausal depression [158] and for its potential to act as a therapeutic for neurodegenerative diseases [159]. In an effort to explore SERMs of ligands that distinguish between ER α/β subtypes, it was discovered that the introduction of a fluorine to carboranyl cages served as effective ERβ-selective agonists, making them useful tools for studying the functions of the ERβ subtype [160].

In recent years, carborane-containing androgen receptor antagonists have also been designed. In 2008, a series of carborane-containing androgen antagonists were synthesized, and in vitro studies found that one of the compounds that had a pyridine ring directly bound to the carborane cage at the 3-position had more potent anti-androgenic activity than the anti-androgen agent flutamide [161]. In 2016, BA321 (Table 6), a novel carborane compound that binds to the androgen receptor (AR) was found to also bind to ERα and ERβ while having no effect on the male sex organs, therefore, acting as a selective androgen receptor modulator (SARM) [162]. BA321 opened the potential for a new therapy option for males with osteoporosis that avoids musculoskeletal mass loss due to androgen deficiency [162]. In 2018, further investigations into SARMs included exploring glycerol and aminoglycerol-containing carboranes as novel androgen modulators [163]. The application of carboranes as steroid analogues was also recently applied to progesterone. This study used a carborane to create a non-steroidal progesterone ligand candidate to treat progesterone-related diseases such as some forms of cancer, without displaying the adverse effects due to cross-activity with other steroid hormone receptors. The carborane derivatives that were synthesized were found to exhibit antagonistic progesterone activity in the submicromolar range (IC_50_ > 0.5 μM) [164].

This series of studies demonstrates that the hydrophobic character of carboranes allow them to act similarly, or in some cases more potently than steroids, while also being able to act as selective ligands.

#### 2.5.5. Therapeutic Applications of Carborane-Modified Nucleosides

Several studies have investigated the use of carboranes to modify nucleosides. In 2010, a study on adenosine that was modified with a para-carborane at the C2′ position was reported [165]. It was found to be an effective platelet inhibitor and to reduce the aggregation, protein secretion, and P-selectin expression induced by thrombin or ADP, making it a promising adenosine analogue for regulating platelet activity [165]. The same group also examined whether the adenosine-carborane cluster analogues would inhibit reactive oxygen species (ROS) production in neutrophils, for inflammatory regulation through adenosine receptor A_2A_ [166]. Other examples include a series of pyrimidines that were modified with a carborane that were studied for their antiviral activity on DNA and RNA viruses and were found to exhibit antiviral activity against Human cytomegalovirus, but not Herpes simplex virus-1, Human parainfluenza virus-3 or Encephalomyocarditis virus [167]. In 2016, adenosine compounds that were modified with a carborane were evaluated for anti-leukemic activity and were found to be less cytotoxic to normal cells than their non-carborane counterparts, while showing higher anti-leukemic potential than purine-based anti-leukemics cladribine, and fludarabine. Furthermore, at concentrations which the carborane compounds elicited high cytotoxicity to leukemic mononuclear cells, normal mononuclear cells experienced low cytotoxicity [168].

#### 2.5.6. Carboranes as Anti-Inflammatory and Anticancer Agents

Carboranes have been found to enhance the anti-inflammatory and anticancer activity of several existing drugs. Cyclooxygenase-2 (COX-2) is a well-known target for anti-inflammatory activity and was the target of several synthesized nonsteroidal anti-inflammatory drug (NSAID) compounds that were modified with a carborane. Preliminary studies had found that ortho-carboranes of indomethacin were active, with meta- and para-carboranes, and adamantane analogues inactive [169]. It was later discovered that the addition of the nido-carborane to indomethacin greatly improved the potency and selectivity towards COX-2 compared to phenyl analogues [170]. While investigating the COX-2 activity of carborane derivatives of NSAID celecoxib, these compounds were found to be cytotoxic against some cancer cell lines [171]. It was discovered that these carborane-celecoxib derivatives do not inhibit COX-2, but rather inhibits the proliferation and induces apoptosis through a caspase-independent pathway [171]. Further studies exploring the COX-2 inhibitor rofecoxib and its carborane derivatives as anticancer agents through COX-2-independent inhibition, found these compounds displayed great selectivity in the micromolar range for melanoma and colon cancer cell lines over normal cells, with the mode of action being dependent on cell type [172]. In 2018, meta-closo-carboranylanilinoquinazoline hybrids were identified as epidermal growth factor receptor (EGFR) inhibitors, with molecular docking analysis showing the carborane moiety interacting with the ATP binding region [173]. The compounds were able to accumulate in the glioma cells, cross the blood brain barrier, and had inherent stability, which make them ideal drugs to treat glioblastoma [173]. Interestingly, another group had synthesized a modified alanine with a carborane side-chain as a substrate for ribosomal incorporation into select peptide sequences that were capable of binding human EGFR, effectively delivering a peptide that was capable of serving as a BNCT agent directly to EGFR-expressing cells [174]. Since EGFR overexpression is associated with several solid tumor types, targeting EGFR with a carborane cluster can result in dual action activity, inhibiting EGFR and acting as a BNCT agent. Carborane-containing compounds have also been explored as selective inhibitors for carbonic anhydrases such as the tumor-associated isoform CAIX over the non-tumor-associated isoform CAII [175], with one compound showing approximately 1230-fold more selectivity for CAIX over CAII [176]. HIF-1α is another anti-cancer target that was found to be inhibited by carboranes, where they prevented hypoxia-induced HIF-1α accumulation without affecting the expression level of HIF-1α mRNA [177]. Lastly, carboranes have been examined as HDAC 2 inhibitors, with an ortho-closo-carborane compound showing potent inhibitory activity against HDAC 2 [178].

#### 2.5.7. Other Novel Applications of Boron Clusters

There is a multitude of miscellaneous applications that have been discovered for incorporating boron clusters, often as a hydrophobic component. A study exploring inhibitors for transthyretin (TTR) had replaced the phenyl ring of flufenamic acid and diflunisal with a carborane moiety, leading to a significant decrease in the NSAID’s COX activity while retaining a similar efficacy as an inhibitor of TTR dissociation [179]. The selectivity was attributed to the carborane moiety, with steric bulk of the carborane preventing π–π stacking that was observed in the COX-2 inhibitors while also filling the binding pocket and maximizing hydrophobic interactions of TTR [179]. An attempt at synthesizing a carborane analogue of aspirin called asborin to inhibit COX-2 was unsuccessful [180]. However, its features were recognized to be ideal for the aldo/keto reductase (AKR) family. Asborin acted as an irreversible inhibitor of AKR1A1, with its bulky size, hydrophobicity, and electron deficiency meeting the requirements for it to act as a candidate for further development [181]. Carboranes have also been explored as potential antimalarials through use of carboranes as phenyl bioisosteres to mimic phenyl groups in current antimalarial drugs. Interestingly, the carborane compounds displayed improved in vitro potency compared to the parent phenyl compound, showing that although the carborane was much larger than the parent phenyl compound, it performed better and that a wide range of bioisosteres should be considered when screening [182]. Metallocarboranes, specifically cobalt-bis-dicarbollides, were designed as lead compounds for the design of HIV protease inhibitors [183]. Carboranes have also been explored as: retinoid receptor antagonists [146,184], as a vitamin D mimetic [185], as antitubercular agents [137], P2XR receptor antagonists to act as antidepressants [186], local anesthetic derivatives of lidocaine called boronicaines [187], and α human thrombin inhibitor [188].

### 2.6. Boron as a Tool to Enhance Drug Efficacy, Delivery, and Targeting

#### 2.6.1. Boron as Protecting Group in Prodrugs

Prodrugs are compounds that have very little to no activity, however, when they undergo an enzymatic or chemical reaction in vivo, they are converted to an active drug. Boron has been used as both the protecting group that is released from the active drug and as the active drug component. The main application of boron as the protecting group of a drug is for targeting cancerous tissue. This is done by exploiting a well-known reaction that occurs between a boronate or boronic acid and a ROS, which results in the formation of an alcohol and a boronate ester or boric acid [189]. Since most cancerous tissues have higher than normal ROS levels compared to normal cells, boron prodrugs can actively target these tissues by releasing an active drug selectively into the cancer cells instead of the normal cells [189]. Additionally, the boronate esters and boric acid that result from this reaction are generally non-toxic to humans [189], making boron prodrugs an ideal tool for creating more effective anticancer drugs.

In recent years, the HDAC inhibitor belinostat has been modified with a pinacol boronate ester-protecting group to serve as a prodrug, with lower efficacy in vitro and higher efficacy in vivo than belinostat [190]. The authors discovered the improved efficacy was due to the accumulation of prodrug in the tumor tissue, resulting in a slow release of the active compound through an ROS-activated mechanism [190]. Further pharmacokinetic studies on the compound (ZL277, Table 7) found that both the oxidative metabolite ZL277-OH-424 and belinostat were glucuronidated by liver microsomes, with ZL227 having a slightly longer half-life and superior bioavailability compared to belinostat [191]. Others have examined using the pinacol boronate ester-protecting group to modify vorinostat as HDAC-inhibiting prodrugs that were activated by H_2_O_2_ [192,193]. The boronic acid-containing prodrugs were able to activate in vitro in the presence of H_2_O_2_ while also effectively enhancing the potency of the chemotherapy drug cytarabine, showing potential to be used as a combination therapy [192]. Boronic acids have also been explored as prodrugs on anticancer agents such as SN-38. Boronic acid derivatives of SN-38 were synthesized as prodrugs, with potency that was greater or comparable to that of SN-38 in vitro and in vivo [194,195]. Another ROS boron prodrug study that was conducted in 2018, was able to synthesize a boron prodrug that incorporated two different drugs, 5-methoxytryptamine and para-benzoquinone methide to be released for therapeutic applications in disease that was related to oxidative stress and cancer [196]. The boron-modified para-benzoquinone methide and 5-methoxytryptamine compounds were connected through a carbamate linker that was inactive at normal physiological ROS levels, but in tissues with a high ROS concentration, would release both active drugs [196]. Gemcitabine (GEM) was also proposed as a boronic ester prodrug that is ROS-activated, converted into GEM in tumor tissue, although there was no increase in efficacy, myelosuppression was reduced [197].

In 2015, ZB497 (Table 7) was synthesized as the prodrug of 4-hydroxytamoxifen (4-OHT) and found to have a 30-fold higher plasma concentration in mice than tamoxifen or 4-OHT, and was more effective at inhibiting the growth of xenograft tumors in mice [198]. Moreover, ZB497 was orally bioavailable, able to be administered at lower concentrations than tamoxifen resulting in a higher therapeutic index, and was able to circumvent metabolism by CYP2D6 to release the active drug. That same year, ZB483 (Table 7) was examined as a boronic prodrug of endoxifen, displaying potent inhibition against breast cancer cell growth both in vivo and in vitro, a similar activation mechanism as ZB497 that is independent of the CYP2D6 enzyme, and is orally bioavailable [199]. Other studies on boronic acid prodrugs with ROS-activation have included modified diarylpropionitrile as ERβ ligands [200] and phosphatidylinositol 3-kinase (PI3K)/mTOR inhibitors [201].

A study that was conducted in 2020 on a methotrexate (MTX) prodrug was found to have similar in vivo efficacy as MTX alone [202]. Although the results were not very significant, this study is important as it highlighted some issues and improvements that needed to be considered to create a more effective ROS-sensitive prodrug treatment using boron. Boronic acids are subject to hydrolysis, which may make it impossible to avoid some non-specific activation due to activation of the prodrugs through an ROS-independent mechanism. This may result in the activation of the drug in other tissues, causing the biodistribution of the prodrug to be unpredictable and may lead to potentially unfavourable pharmacokinetics [202]. Considering both the success and the possible issues that may surround utilizing an ROS-activated boron prodrug approach, further consideration to possible undesirable pharmacokinetic properties and off-target effects should be kept in mind.

#### 2.6.2. Prodrug-Modified Boron Compounds

In addition to being used as ROS-activated prodrugs, boron-containing drugs have also been modified to form a prodrug, with the intention of increasing the favourability of their pharmacokinetic properties. The two FDA-approved peptidic boronic acid drugs, ixazomib and bortezomib, are given in prodrug form as ixazomib citrate and bortezomib mannitol ester. The addition of mannitol and citrate serve to increase the solubility while also protecting the active boronic acid component. In the case of bortezomib, the mannitol ester-protecting group results in reduced toxicity as bortezomib is only released when in low pH tumor environments [203], however bortezomib still has undesired toxicity. Thus, a new protecting group has been proposed to alleviate some of the toxicity that is associated with bortezomib. BORSA, (Table 7) a prodrug of bortezomib with a sialic acid (SA)-protecting group was designed and was found to reduce non-specific toxicity in vivo, while showing improved efficacy in vitro [203]. Recently, there were attempts at synthesizing a library of novel dipeptide boronic acid inhibitors that were similar to bortezomib but with substitutions at the 3 α-carbon positions of the peptide backbone and various protecting groups at the boronic acid site [204]. Several of the compounds were found to be comparable to bortezomib in potency in vitro and in vivo pharmacokinetic results in rats and xenograft mice showed potential, however further optimization on oral bioavailability is needed [204]. Prodrugs of carborane BNCT therapy agents have also been explored to improve the poor solubility of the highly hydrophobic carborane moiety. Several promoieties have been proposed that increase solubility before undergoing hydrolysis to release the active compound, with amino acid based promoieties showing improved solubility with good rates of hydrolysis in cerebrospinal fluid [205]. These studies show that although some boron drugs may have undesirable pharmacokinetic properties such as low solubility, non-specific toxicity, and low bioavailability or low specificity, these can be improved on as boron can act as a site for modification through the addition of a protecting group to form a prodrug.

#### 2.6.3. Boron in Softdrugs

While prodrugs are designed to limit the toxicity through specific delivery, softdrugs limit the toxicity through the deactivation of the drug once it leaves the site of action and goes into systemic circulation. Typically, softdrugs should undergo rapid deactivation through predictable mechanisms that are integrated into the drug design process, ideally through hydrolytic deactivation [206]. Due to this property, softdrugs are typically topical and are introduced directly to the target site.

**Figure 4:**
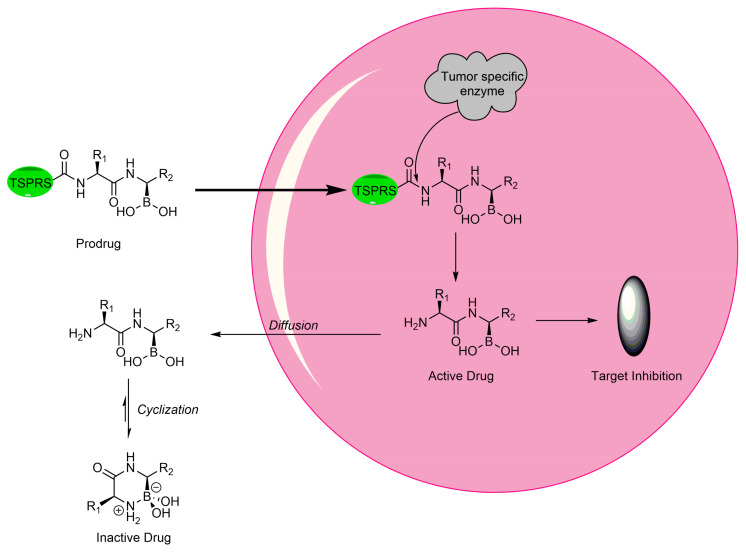
Pro-softdrug boronic acid inhibitor-targeting tumor cells. Tumor-specific protease recognition sequences (TSPRS) are recognized by tumor-specific proteases that cleave TSPRS, releasing the active drug inside the tumor cell. The drug will go on to inhibit the target, before slowly diffusing outside of the tumor cell. The physiological pH outside the tumor cell causes the drug to undergo cyclization, deactivating it [208].

In 2010, a study using the softdrug approach to create benzoxaborole PDE4 inhibitors for topical application had one of the compounds, 5-(5-ethoxycarbonyl-2-pyridyloxy) benzoxaborole, showing broad anti-inflammatory activity and converted to the inactive acid rapidly in vitro and in vivo in mice [207]. Additionally, it had a high subcutaneous bioavailability, while having a low oral bioavailability, showing that it is safer than a common PDE4 inhibitor, rolipram [207]. A unique study that was conducted in 2011 incorporated both the prodrug and softdrug method in creating dipeptidic boronic acid proteasome inhibitors as potential highly selective tumor-targeting drugs [208]. The prodrug aspect involves attaching the dipeptidyl drug to the C-terminus of tumor-specific protease recognition sequences (TSPRS), which are only recognized by tumor-specific proteases that cleave them releasing the softdrug directly into the tumor cell (Figure 4) [208]. The softdrug aspect was designed using previous reports that dipeptidyl boroPro undergoes pH-dependent cyclization at physiological pH due to deprotonation of the N-terminal amine, which acts as a nucleophile and attacks the boronic acid to induce cyclization, whereas in low pH environments such as tumors, the active open chain form of the drug is favoured [209]. This allows the drug to get cleaved and activated in the tumor, while rapidly undergoing cyclization and deactivation upon diffusion outside of the tumor cell. The study candidate, a dipeptide of boroLeu was potent, cytotoxic, and resistant to degradation by cellular proteases making it a good candidate for further development [208]. The utilization of softdrugs for designing boron-containing compounds is very promising and shows that this approach can be incorporated with other methods to improve the safety of topical boron-containing drugs.

#### 2.6.4. Boron in Micelle Formation as Nanocarriers

Nanocarriers are nanomaterial, such as liposomes and micelles, that are used as a transport carrier within the body. Previously, carborane liposomes and vesicles as nanocarriers were discussed for BNCT. There are other examples of nanocarriers that have been studied for their use in BNCT and in the delivery of other boron-containing drugs. In 2014, liposomal bortezomib nanoparticles were synthesized by incorporation into the liposome using surface conjugation [210]. Although there were some issues regarding the amount of bortezomib that could be loaded and the particle stability, it was able to inhibit the proteasome and in vivo studies, showed that it was able to significantly reduce tumour growth and reduce systemic toxicity compared to free bortezomib [210]. A recent study that was conducted in 2018 targeting bone cancer involved a micelle-based nanocarrier, in which polyethylene glycol (PEG)-bound alendronate is used to target bone tumor tissue and PEG-bound catechol to bind bortezomib to form the micelle nanoparticle. This method functionalizes the interaction of boronic acid with the 1,2 diol on catechol to form a pH-sensitive boronate ester, effectively neutralizing the boronic acid, and anchoring it to the PEG-base micelle nanoparticle until it reaches its target. Upon reaching an acidic tumor environment, the catechol boronate ester becomes hydrolyzed releasing bortezomib, with results showing reduced systemic toxicity and improved therapeutic effects in vitro and in vivo [211]. Using a similar mechanism, another study used a boronate ester-linked PEG co-polymer that was bound to γ-camptothecin-glutamate N-carboxyanhydride, that allowed for self-assembly into an amphiphilic micelle nanocarrier that was used to encapsulate doxorubicin. The dual prodrug micelles allowed for the release of both drugs upon hydrolysis of the boronate ester at low pH tumor environments. In vitro examination found the micelles maintained their structural integrity in water, were able to inhibit HepG2 cell proliferation, and in a simulated tumor environment, showed efficient boronate ester cleavage and cellular entry [212].

#### 2.6.5. Phenylboronic Acid Nanoparticles

Phenylboronic acid (PBA) nanoparticles have been extensively studied in various forms for drug delivery including nanogels and as self-assembling vesicles. PBA-functionalized silica nanoparticles have been studied for their ability to accommodate many drugs and for the PBA moiety to enable the nanoparticle to have a glucose-responsive regulated drug release [213]. In the case of diabetes mellitus, insulin can potentially be loaded into these glucose-sensing micelle nanoparticles that are held together due to the amphiphilic nature of the PBA-polymer. Upon binding of the PBA to glucose, formation of a hydrophilic phenylborate is formed, shifting the equilibrium balance from amphiphilic to hydrophilic and resulting in micelle collapse and the release of insulin [213]. Other similar PBA-based carriers have been examined, with in vivo results showing faster insulin release following a glucose challenge compared to injectable insulin, and reduced hypoglycemic index when administered during normoglycemia in mice [214]. Even though these examples are promising novel approaches to utilizing PBA, there are still some issues that need to be addressed. The effective encapsulation of insulin is difficult due to the lack of strong interaction between insulin and the capsulation molecules, while the effective and quantitative release of insulin on demand by PBA nanoparticles is still rare [213]. Nanoparticles are also difficult to design for oral administration due to poor absorption of nanocarriers into intestinal mucosa, reducing its ability to reach systemic circulation [213]. Nonetheless, PBA nanoparticles as a drug delivery mechanism are still a relatively new concept, but definitely warrant further investigation.

#### 2.6.6. Boron in Dendrimers and Porphyrins

Dendrimers are globular, branched macromolecules that are designed as drug carriers. Dendrimers are water soluble and have a uniform composition, are able to incorporate functional groups into the periphery or interior, and are highly versatile, allowing control over the number of drugs the dendrimer can carry [141,215]. Carboranes that are incorporated onto dendrimers have been delivered to tumor tissues for anticancer treatment with in vitro studies finding significant boron concentrations achieved when boron-containing dendrimers targeted the epidermal growth factor receptor [141]. In addition to dendrimers, porphyrins have also been examined as carriers for boron delivery. Porphyrins that are loaded with boron were proposed as potential BNCT agents and were able to be administered into melanotic melanoma tumors, which are known to be aggressive and radioresistant [141].

#### 2.6.7. Utilizing Boron-Carbohydrate Chemistry for Drug Delivery

**Figure 5:**
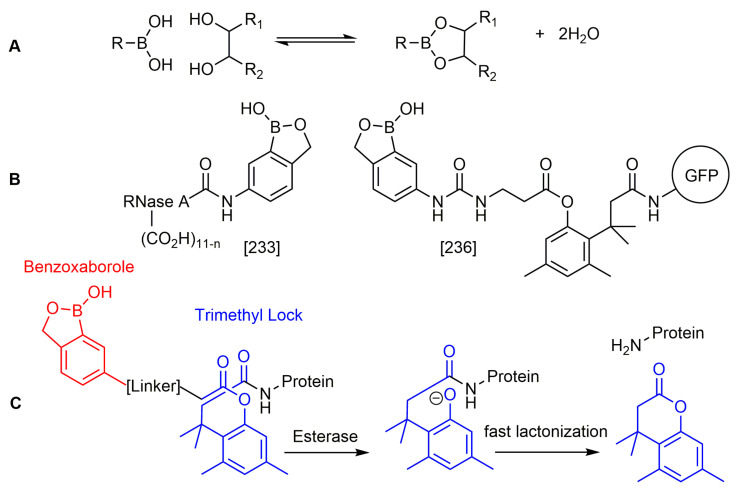
(**A**) Boronic acids bind to 1,2 and 1,3 diols on carbohydrates forming a cyclic boronic ester. (**B**) Benzoxaborole-modified proteins as a delivery mechanism to transport proteins into cell. RNaseA [217] and green fluorescent protein (GFP) [220] were used as proof of concept. (**C**) Benzoxaborole-trimethyl lock protein shuttling mechanism. Benzoxaborole binds to carbohydrates on the cell surface and the entire molecule is internalized by endocytosis. Benzoxaborole is cleaved by a cellular esterase triggering trimethyl lock that undergoes rapid lactonization, releasing unmodified protein [220].

Boronic acids, particularly benzoxaboroles have also been found to act as a deliverer of drugs by exploiting their ability to reversibly bind with 1,2 and 1,3 diols (Figure 5A). These diols are commonly found on carbohydrates, with some carbohydrates having a role in many pathological and physiological processes including the development of cancer [216]. This characteristic has been the focus of studies in which boron-mediated delivery systems were designed. For example, in 2012 a study investigated the potential for the boronic acid carbohydrate interaction to mediate cytosolic delivery of a cytotoxin (RNase A) through endocytosis. The study proposed using an RNase A that was bound to a benzoxaborole due to its affinity to bind to sialic acid, a carbohydrate that is frequently found on the glycocalyx of cancer cells [217]. The saccharide-boronate complex undergoes endocytosis, with endosome pH decreasing over time and lowering the affinity of boronates to sugars that results in disassociation of the saccharide-boronate complex [218]. The free boronate-RNase A molecule is more hydrophobic and thus eases its translocation from endosome into the cytosol [219]. It was found that the boronation of RNase A increased cellular uptake four- to five-fold and inhibited the proliferation K-562 cells (IC_50_ 4.1 µM) compared to unmodified RNase A (IC_50_ > 50 µM) and chemically-inactivated boronated RNase A (IC_50_ > 50 µM), which suggests successful delivery to the cytosol [217]. One of the major drawbacks to this design is the irreversible modification of protein carboxyl groups to directly attach the benzoxaboroles, which compromised the cytoxicity of the boronated RNase A [217]. Considering this, the authors designed a bioreversible benzoxaborole modification that, when applied to proteins, could facilitate transport of the protein into the cell before the benzoxaborole linker is cleaved by esterases that are abundant in the cytosol [220]. The design utilizes a trimethyl lock scaffold and is attached to the protein via a *N*-hydroxysuccinimide ester that selectively binds to amino groups on the N-terminus or the ε-amine of lysine residues [220]. Once the molecule is internalized into the cell, an esterase cleaves the benzoxaborole from the trimethyl lock triggering rapid lactonization and release of the native protein in its original state. These studies show that utilizing boron’s ability to reversibly bind with diols is a notable characteristic that can be exploited to design specific delivery agents.

## 3. Conclusions

Initially studied for its application in BNCT, boron has received an increased focus in recent decades with the creation of novel therapeutics, protecting groups, and drug carriers in medicinal chemistry. Although the search to find an effective delivery method of boron to tumor cells continues, the scope of boron applications has shifted with boron incorporated into structures such as diazaborines as antimicrobials, peptidic boronic acids as proteasome inhibitors for cancer therapy, benzoxaboroles as LeuRS inhibitors, and cyclic boronates as β-lactamase inhibitors for microbial infections. Boron clusters, originally proposed for BNCT applications, have recently gained traction with new applications that exploit their properties such as 3D aromaticity, electron deficiency, and ability to act as hydrophobic, amphipathic, or hydrophilic molecules, depending on the particular configuration. Their applications are numerous and have included acting as steroidal receptor agonists/antagonists and as anti-inflammatories among others. Recently, boron applications have extended further, with boronic acids/esters utilized as the protecting group in anticancer prodrugs that activate in high ROS tumor environments, and as potential nanocarriers to transport drugs across the cell membrane. While boron-focused drug design is still a relatively new field, current discoveries and applications have demonstrated boron as a viable candidate with considerable potential for future development.

## Figures and Tables

**Table 1:** Diazaborine medicinal agents.

Diazaborine Structure	Name	Reference
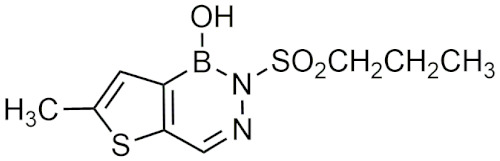	1,2-dihydro-1-hydroxy-6-methyl-2-(propanesulphonyl)-thieno (3,2-D) (1,2,3)-diazaborine (Sa 84.474)	[25]
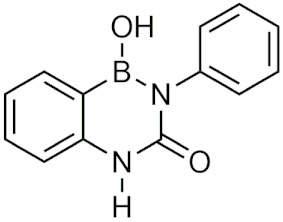	1,2-dihydro-1-hydroxy-2-phenyl-2,4,1-benzo[e]diazaborin-3(4H) -one	[27]
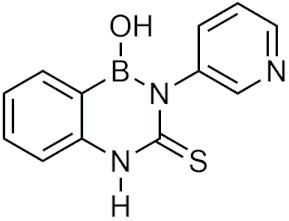	1,2-dihydro-1-hydroxy-2-(3-pyridyl)-2,4,1-benzo[e]diazaborin-3(4H)-thione	[27]
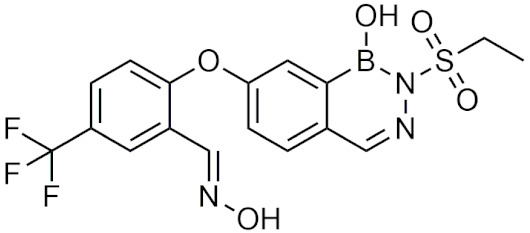	AN12855	[28]

**Table 2:** Peptidic boronic acid medicinal agents.

Peptidic Boronic Acid Structure	Name	Reference
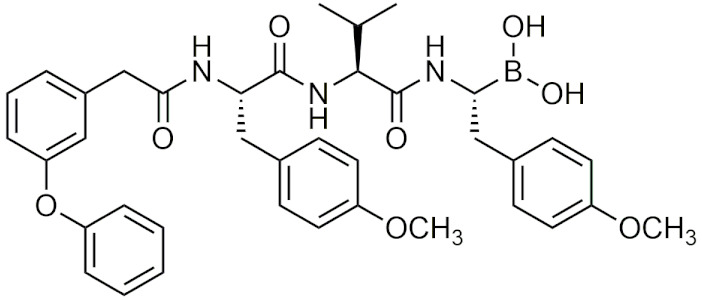	AS-06	[46]
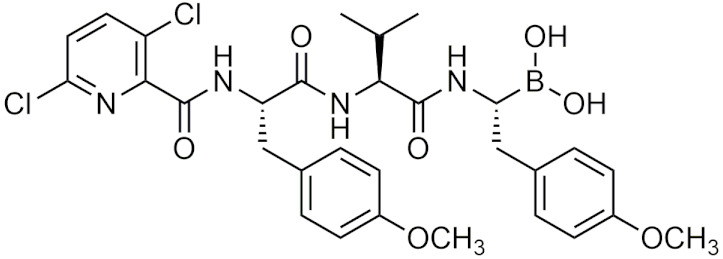	AS-29	[46]
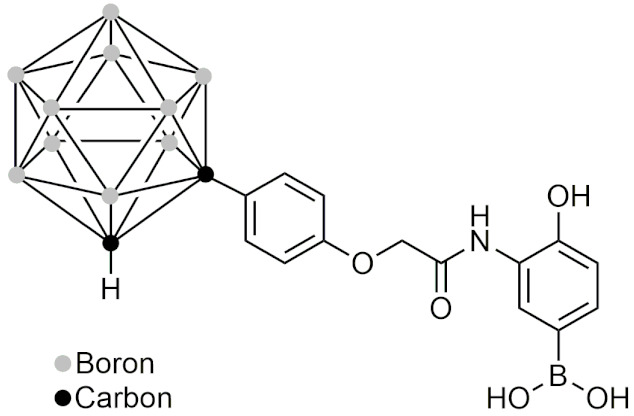	GN26361	[54]
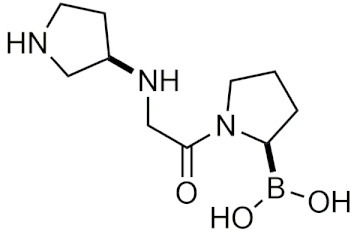	PHX1149 (dutogliptin)	[4]
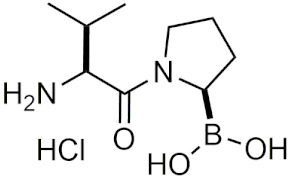	PT100 (Talabostat/Val-boroPro)c	[4]
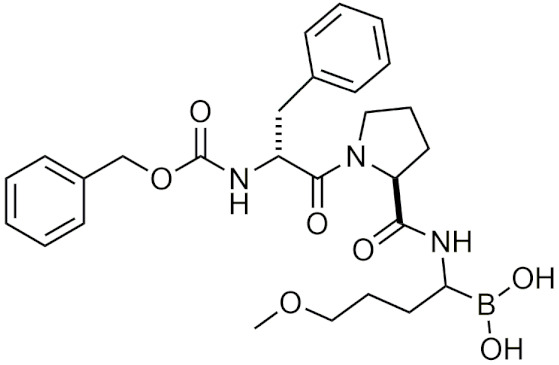	TRI50c	[4]
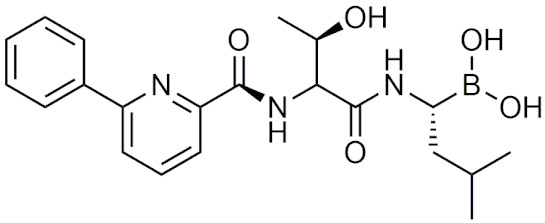	CEP-18770 (delanzomib)	[64]

**Table 3:** Benzoxaborole medicinal agents.

Benzoxaborole Structure	Name	Reference
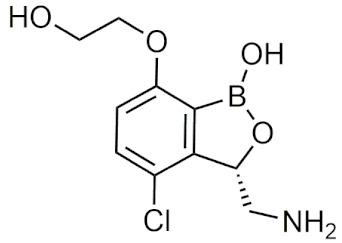	GSK3036656	[71,74]
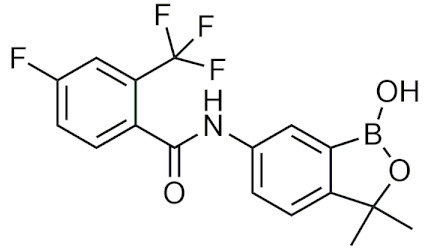	Acoziborole	[77]
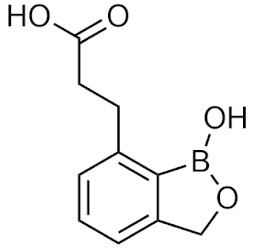	AN3661	[84]

**Table 4:** Benzoxaborole medicinal agents.

Benzoxaborole Structure	Name	Reference
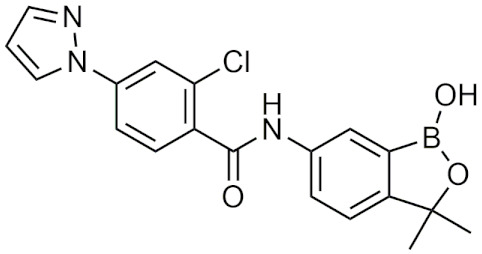	AN7973	[102]
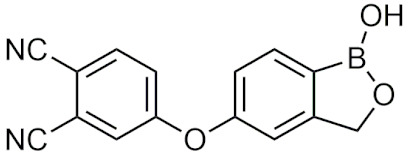	AN2898	[103]
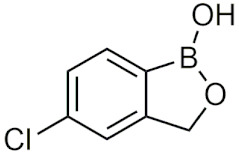	AN2718	[104]
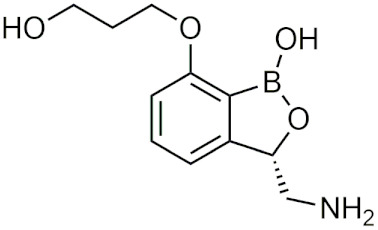	AN3365 (GSK2551052/epetraborole)	[6]

**Table 5:** Cyclic boronic acid as β-lactamase inhibitors and anti-inflammatory drugs.

Cyclic Boronic Acid Structure	Name	Reference
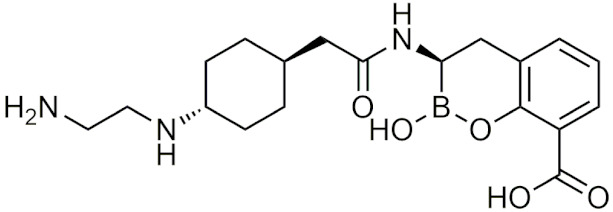	Taniborbactam (VNRX-5133)	[120]
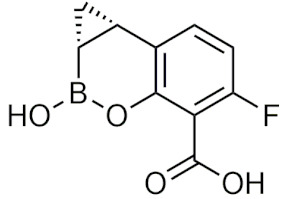	QPX7728	[121]
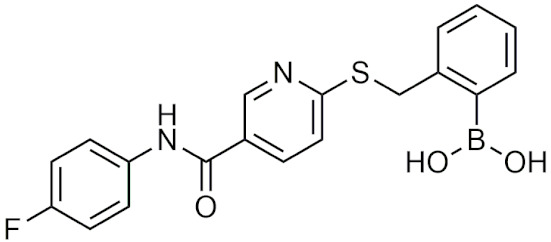	SX-517	[126]
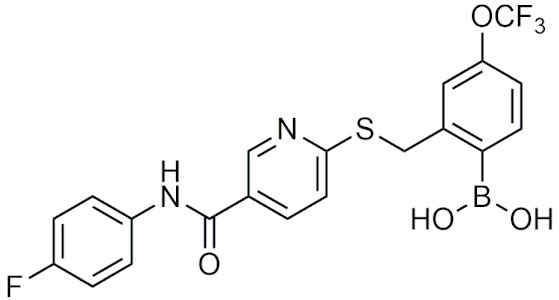	SX-576	[127]

**Table 6:** Carboranes as steroid agonists/antagonists and carborane-modified nucleosides.

Carborane Structure	Name	Reference
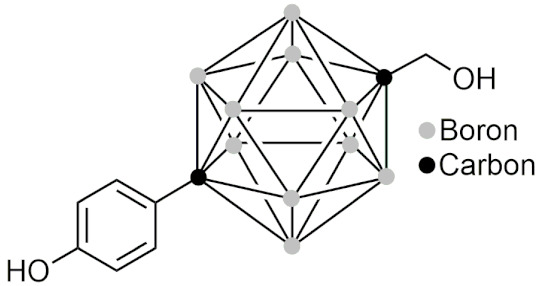	BE120	[156]
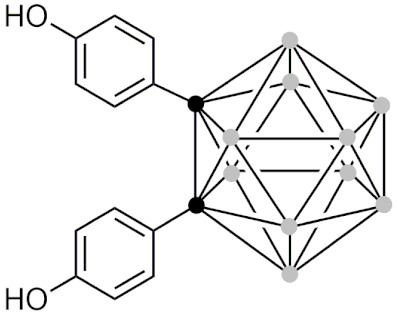	BE360	[157]
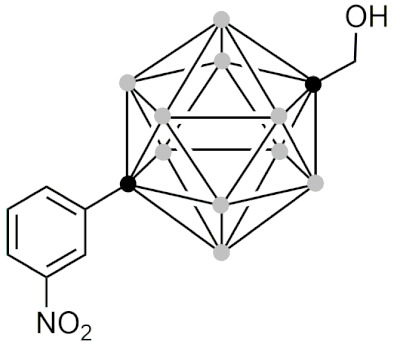	BA321	[162]
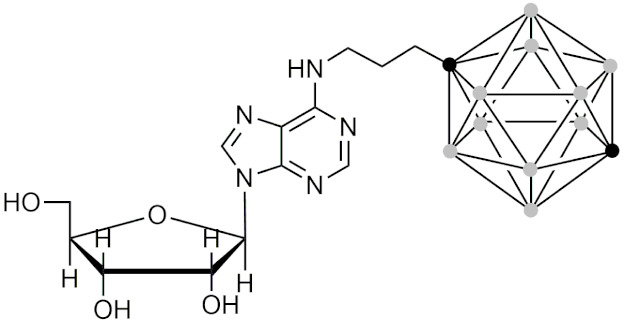	6-N-[(1,12-dicarba-closo-dodecaboran-1-yl)propan-3-yl]adenosine	[166]
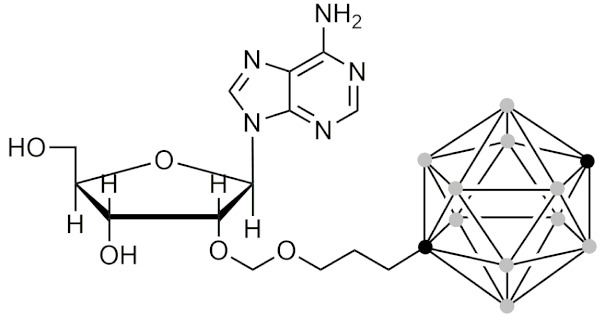	2’-O-[(1,12-dicarba-closo-dodecaboran-1-yl)propyleneoxymethyl]adenosine	[166]

**Table 7:** Boronic acid prodrugs with the prodrug moiety in blue and the active drug in black.

Boronic Acid Prodrugs	Name	Reference
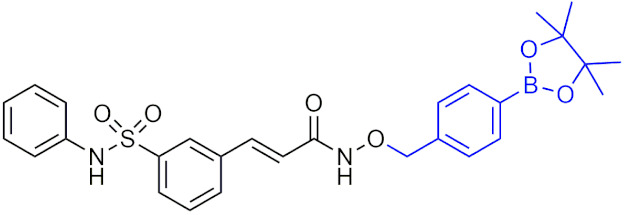	ZL277 (belinostat prodrug)	[191]
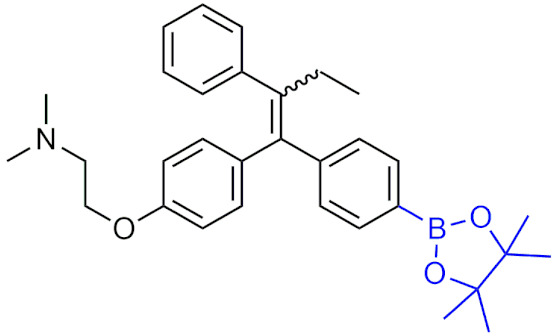	ZB497 (4-hydroxytamoxifen prodrug)	[198]
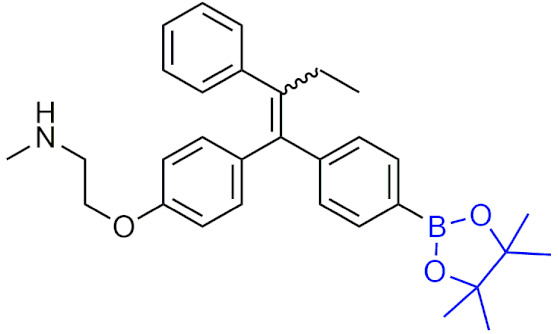	ZB483 (Endoxifen prodrug)	[199]
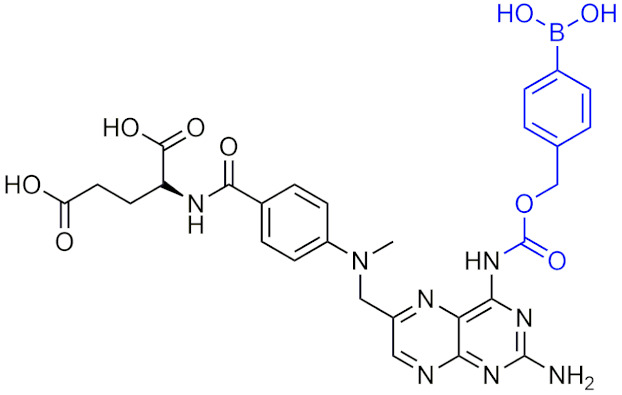	(4-(((2-amino-4-((((4-boronobenzyl)oxy)carbonyl)amino)pteridin-6-yl)methyl)(methyl)amino)benzoyl)-D-glutamic acid (methotrexate prodrug)	[202]
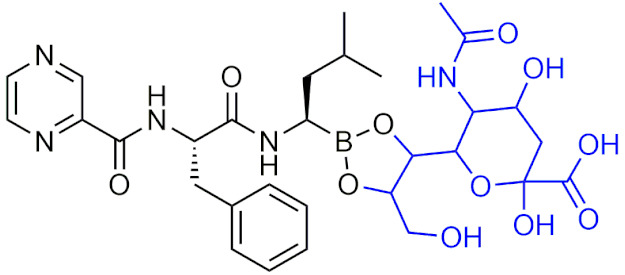	Bortezomib-sialic acid (BORSA) (Bortezomib prodrug)	[203]

## Data Availability

Data sharing not applicable.
